# A novel type of biochar from chitinous *Hermetia illucens* waste with a built-in stimulating effect on plants and soil arthropods

**DOI:** 10.1038/s41598-023-35460-6

**Published:** 2023-05-23

**Authors:** Piotr Bulak, Kinga Proc-Pietrycha, Monika Kaczor, Katarzyna Złotko, Cezary Polakowski, Dariusz Wiącek, Hanna Waniak-Nowicka, Emil Zięba, Adam Waśko, Patryk Oleszczuk, Andrzej Bieganowski

**Affiliations:** 1 Institute of Agrophysics, Polish Academy of Sciences, Doświadczalna 4, 20-290 Lublin, Poland; 2 Analytical Laboratory, Faculty of Chemistry, Institute of Chemical Science, Maria Curie-Skłodowska University, M. Curie-Skłodowska Square 3, 20-031 Lublin, Poland; 3 Department of Biomedicine and Environmental Research, Faculty of Medicine, Institute of Biological Sciences, The John Paul II Catholic University of Lublin, Konstantynów 1J, 20-708 Lublin, Poland; 4 Department of Biotechnology, Microbiology and Human Nutrition, Faculty of Food Science and Biotechnology, University of Life Sciences in Lublin, Skromna 8, 20-704 Lublin, Poland; 5 Department of Environmental Chemistry, Faculty of Chemistry, Maria Skłodowska-Curie University, Maria Curie-Skłodowska Square 3, 20-031 Lublin, Poland

**Keywords:** Element cycles, Environmental impact, Entomology

## Abstract

The breeding of insects generates waste in the form of insect excrement and feed residues. In addition, a specific chitinous waste in the form of insect larvae and pupae exuvia is also left. Recent research tries to manage it, e.g., by producing chitin and chitosan, which are value-added products. The circular economy approach requires testing new, non-standard management methods that can develop products with unique properties. To date, the possibility of biochar production from chitinous waste derived from insects has not been evaluated. Here we show that the puparia of *Hermetia illucens* are suitable for biochar production, which in turn exhibits original characteristics. We found that the biochars have a high nitrogen level, which is rarely achievable in materials of natural origin without artificial doping. This study presents a detailed chemical and physical characterization of the biochars. Moreover, ecotoxicological analysis has revealed the biochars’ stimulation effect on plant root growth and the reproduction of the soil invertebrate *Folsomia candida*, as well as the lack of a toxic effect on its mortality. This predisposes these novel materials with already built-in stimulating properties to be used in agronomy, for example as a carriers for fertilizers or beneficial bacteria.

## Introduction

In recent years, the industrial usage of insects for feed and food production has been increasing, especially in areas where insects are not traditionally eaten^1^. In Europe, upon the entry into force of Commission Regulation (EU) 2017/893, the European Union allowed insects and insect proteins to be used in the production of aquaculture animal feed for the first time. Insect species that fulfill the safety requirements set out in the above regulation include *Acheta domesticus* (Linnaeus, 1758), *Alphitobius diaperinus* (Panzer, 1797), *Gryllodes sigillatus* (Walker, 1869), *Gryllus assimilis* (Fabricius, 1775), *Hermetia illucens* (Linnaeus, 1758), *Musca domestica* (Linnaeus, 1758) and *Tenebrio molitor* (Linnaeus, 1758)^2^. Two later EU Commission Regulation implementation approved *Locusta migratoria* (Linnaeus, 1758)^3^ and *T. molitor*^4^ as novel foods for humans. Now, also *A. domesticus* and *A. diaperinus* have also been allowed.

The legislative changes will facilitate the faster development of new branches in the food and feed industries based on the production of insects. Insect excrement combined with feed remnants, which frequently contain deceased insects, will be produced in greater quantities as the use of insects in food production increases. These wastes are usually sold as plant fertilizers^5^ and they can also be employed as a substrate in biogas generation^6,7^. Holometabolic insects also produce a chitinous type of waste—puparia, also called pupal exuviae, which are the exoskeleton of pupae. This waste remains after the emergence of the adult form of the insect. Typically, puparia are discarded with all the other post-production wastes or eaten by insects in the younger stages of development (based on our experience with *H. illucens* breeding). However, in some types of breeding systems for certain varieties of insects, they can be easily gathered. This is the case for *H. illucens*, where large quantities of puparia are produced during breeding. There are indications of the usefulness of this type of waste in chitin production^8^.

Another possibility, which has not yet been researched, is the use of puparia in production of biochar, which might be regarded as a novel method of valorizing this kind of waste. Biochar or biocarbon is produced by the thermal degradation (pyrolysis) of organic matter such as plant material or biowastes (sewage sludges or biogas residues)^9^. Biochar can be used as an adsorbent in chemical and industrial processes and can act as a soil amendment, that influences soil carbon sequestration, greenhouse gas (GHG) emissions, water–air conditions, as well as plant growth^10^.

*H. illucens* (the black soldier fly—BSF) is an insect from the order Diptera (Stratiomyidae), which is present across the northern and southern hemisphere. In recent years, interest in this insect has been growing, due to its interesting properties and variety of uses. It can be employed in the utilization and cleaning of various waste organic matter in a process called entomoremediation^11–15^. Studies^16–19^ have shown that it can also be used as feed for other animals, as well as in biodiesel production, and can act as a source of new antimicrobial compounds^20^.

To the best of our knowledge, there are no publications that discuss the utilization of chitinous waste materials derived from insects in biochar production. The production and application of biochar from isolated shrimp chitin as a dye absorbent were recently investigated by Zazycki et al.^21^. Despite showing good decolorization properties, the production of biochar from chitin has underlying disadvantages, including its cost and environmental impact (aggressive chemicals are employed during the extraction of chitin). The puparia of *H. illucens* contain chitin, and the production of biochar directly from them would minimize the extraction costs and theoretically allow for maintaining the positive sorption properties of chitin and transferring them to the biochar. Moreover, it indicates a new way of managing chitinous waste material and would not reduce the pool of pure chitin from marine sources available on the market, which should instead be used in the development of more valuable products (e.g., chitin wound dressings)^22^. With reference to humans’ aggressive management of sea and ocean resources (overfishing) and their devastating pollution, the direct use of chitinous insect waste for biochar production, without previous extraction of chitin, should also transpire to be more environmentally friendly than that of the chitinous parts of krill or shrimp.

Here, we produced and characterized biochar derived from chitinous insect waste material—the puparia of *H. illucens*. In addition to the physicochemical properties, an in-depth analysis of the content of the contaminants potentially occurring in biochars (heavy metals and polycyclic aromatic hydrocarbons (PAHs)) as well as the toxicity of the biochars was evaluated using various biotests (Collembola reproduction test, Phytotoxkit, Microtox).

## Materials and methods

### Feedstock material

Puparia from *H. illucens* pupae were chosen as the insect material for biochar production. The *H. illucens* larvae were reared in groups in the laboratory of the Institute of Agrophysics of the Polish Academy of Sciences in Lublin (Poland) in a container made from plexiglass (86 cm × 53 cm × 46 cm). The number of larvae in the container was in the range of 1500–2000 individuals. The culture conditions were: a temperature of 27 ± 1 °C with a substrate humidity of 50–80% in darkness. The larvae were grown on coconut fiber and fed commercial carp fish (manufacturer: FloraZoo, Chełmża, Poland) feed with the following composition: 54.80% carbohydrates, 25.00% protein, 5.00% fat and oil, 5.80% crude fiber, 5.70% ash, 1.25% lysine, 1.00% calcium, 0.97% phosphorus and 0.40% methionine (percentages are given on the basis of dry weight (DW)). In these conditions, the larvae transformed into pupae after 16 days. The pupae crawled out of this container looking for dry places to metamorphose and were then collected and transferred to the insectarium, where the flies emerged. For further biochar production, puparia from *H. illucens* breeding were taken as they were, without any purification stage. The skipping of the purification stage was related to the fact that the puparia were only lightly contaminated with the substrate, and omitting any additional treatments before pyrolysis may be advantageous economically.

### Pyrolysis conditions

**Figure 1:**
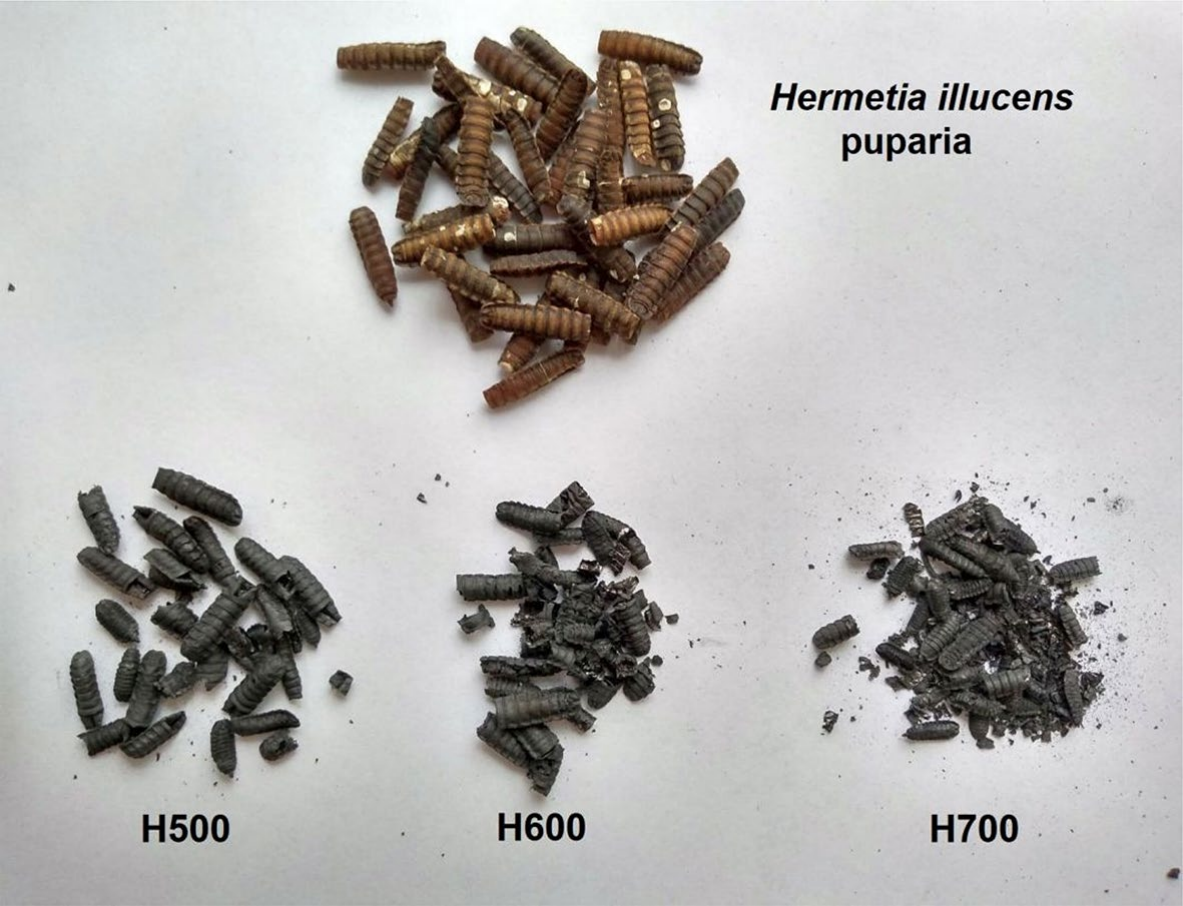
The appearance of *Hermetia illucens* puparia and biochars obtained from it.

The pyrolysis was carried out in a modified laboratory furnace (L15/12, LAC, Czech Republic) with a gas feed. The air-dried puparia were placed in quartz tubes and inserted into the furnace. Before the heating was started, the air inside the furnace was replaced by blowing in nitrogen (99.99999% purity) for 1 h from a generator (Zefiro 5HP, Cinel, Portugal) with a flow rate of 5 l min^−1^. The puparia were pyrolyzed for 30 min at three temperature variants: 500 °C, 600 °C, and 700 °C. The nitrogen flow was constant during the pyrolysis process. When the temperature in the furnace was equal to the ambient temperature and the samples had cooled, the nitrogen flow was stopped and the biochars were removed from the furnace. The biochars were labeled as H500, H600, or H700. Figure 1 shows the structural characteristics of the pristine material and the produced biochars. The biochar from *H. illucens* produced at all the temperatures retained the macroscopic structure of puparia.

### Physicochemical analysis

The pH of the biochars was measured in distilled water at a ratio of 1:20 (w/v) after shaking the samples for 1.5 h at 300 rpm (HD40d multi, HACH, CO, USA). The DWs of the samples were determined after drying at 105 °C for 24 h. The volatile solids (VS) and ash content were analyzed after burning the samples in a muffle oven at 550 °C for 2 h. A porosimetric analysis was carried out using a Micromeritics Accelerated Surface Area and Porosimetry System (ASAP 2420) analyzer (Norcross, GA, USA). The samples were degassed at 200 °C to reach a stable pressure of 0.005 mm Hg in glass tubes. They were then placed in liquid nitrogen, with gaseous nitrogen being dosed (adsorption) until a specific P/Po pressure was obtained, according to the measurement points previously programmed and designed to form an adsorption curve. During the desorption, the nitrogen introduced into the pores of the analyzed samples was eliminated by reducing the pressure in the glass tubes. The specific surface area (SSA) of the biochars was calculated on the basis of the Brunauer–Emmett–Teller (BET) equations (multilayer adsorption). The dynamic gravimetric water sorption was measured by a Dynamic Vapor Sorption (DVS) Intrinsic analyzer (SMS, United Kingdom) at 20 °C. The content of C, H, and N was determined on a CHN 2004 analyzer (Perkin Elmer, USA). The total O content was determined by subtraction, as follows^23^:

1 $${\text{O }}\left( {{{\% }}\frac{{\text{w}}}{{\text{w}}}} \right) = 100 - {\text{Ash }}\left( {{{\% }}\frac{{\text{w}}}{{\text{w}}}} \right) - {\text{C }}\left( {{{\% }}\frac{{\text{w}}}{{\text{w}}}} \right) - {\text{H }}\left( {{{\% }}\frac{{\text{w}}}{{\text{w}}}} \right) - {\text{N }}\left( {{{\% }}\frac{{\text{w}}}{{\text{w}}}} \right) - {\text{S}}\left( {{{\% }}\frac{{\text{w}}}{{\text{w}}}} \right)$$

Total S content was determined by ICP-OES analysis (details below). The Fourier transform infrared spectroscopy (FT-IR) spectra of the samples were recorded using a Nicolet 6700A FT-IR spectrometer (Thermo Scientific) equipped with diamond attenuated total reflectance (ATR). The absorbance values were evaluated between 400 and 4000 cm^−1^. Each spectrum resulted from 128 scans on one replication. The spectra were averaged from five replications^24^ with SpectraGryph 1.2 software (Dr Friedrich Menges, Germany).

The scanning electron microscopy (SEM) of the surface morphologies of the samples was taken with a Carl Zeiss AG Ultra Plus (Oberkochen, Germany) at a magnification range between 150× and 50,000× with no coating. The micrographs were made using the high-vacuum secondary electron detection technique. Energy-dispersive X-ray spectroscopy (EDX) photography was achieved with an energy-dispersive spectroscopy (EDS) detector (Brucker, Billerica, MA, US) mounted in the SEM, with a resolution of 123 eV and beam energy of 20 kV. The sample scanning time was 300 s (real time).

The X-ray diffraction (XRD) patterns of the biochars were analyzed with Co Kα radiation (λ = 1.78901 nm) using an Empyrean instrument (Malvern PANalytical, UK) operated at 40 kV and 25 mA. A nickel filter was utilized to absorb the Kβ Co X-rays on the incident beam. The diffraction pattern was recorded with a 0.008° s^−1^ scanning rate, 0.02° 2θ step size, and 2.5 s time per step.

### Elemental content

The mineralization of the biochar samples was carried out in a solution of HNO_3_ + HCl in proportions of 3:1 + 2 ml HF/sample. The element content was determined using an inductively coupled plasma-optical emission spectroscopy (ICP-OES) system (Thermo Scientific iCAP Series 6500) in accordance with Bulak et al.^25^. The following wavelengths (nm) were employed for the determination of elements: Al 396.152, As 189.042, Ba 493.409, Be 265.045, Ca 184.006, Cd 226.502, Co 228.616, Cr 267.716, Cu 324.754, Fe 261.187, Ga 417.206, Hg 184.950, K 766.490, Li 670.784, Mg 285.213, Mn 259.373, Mo 204.598, Na 589.592, Ni 231.604, P 178.284, Pb 220.353, S 180.731, Sc 361.384, Se 196.090, Sr 421.552, V 310.230 and Zn 206.200. The internal standard of Y was added to each sample at a concentration of 5 ppm.

### Heavy metal sorption experiment

Stock solutions of Ni^2+^, Cd^2+^ and Pb^2+^ were prepared by dissolving Ni(NO_3_)_2_·6H_2_O, Cd(NO_3_)_2_·4H_2_O and Pb(NO_3_)_2_ in deionized water (HLP_10_, Hydrolab, Poland). HNO_3_ or NaOH were used to adjust the pH 5 of the final solutions. The concentration of metal ions in the final solution was 100 mg l^−1^. Each examined biochar sample (100 mg) was mixed with 10 ml of each of the metal ion solutions and agitated at 250 rpm at room temperature (22 ± 2 °C) for 24 h. The biochars were then separated from the background solution by centrifugation at 12 000 rpm for 5 min^26^. The metal content in the background solution was measured with an ICP-OES (Thermo Scientific iCAP Series 6500).

### Polycyclic aromatic hydrocarbon (PAH) content

The freely dissolved (C_free_) PAH content was determined in water suspensions^27^. One gram of biochar (DW) ground with a mortar and pestle was shaken in glass flasks (50 ml) with 0.35 g polyoxymethylene (POM) and 40 ml Milli-Q water (0.2 g l^−1^ NaN_3_ was employed as a biocide). After 28 days, the POM strips were removed from the solution, wiped off with tissue paper, and extracted using heptane:acetone (20 ml, 4:1, v/v) by horizontal shaking (48 h). Deuterated 16 PAHs in isooctane (180 ng each, internal standard) were added before extraction. An aliquot of 20 ml of the organic phase (after the POM extraction) was evaporated to 1 ml on a rotational vacuum concentrator (RVC 2-25CD plus, Martin Christ, Germany). The extract was transferred to a gas chromatography (GC) vial and analyzed with gas chromatography–mass spectrometry (GC–MS).

The determination of the organic solvent-extractable PAH content (C_tot_) included the extraction of samples via the Soxhlet method with toluene (125 ml) for 24 h at 160 °C (sample weight 1 g DW), followed by the cleaning up of the concentrated extracts using dimethylformamide (DMF)/hexane as described by Brändli et al.^28^. The re-collected phase was reduced and applied to an open micro glass column (150 mm × 7 mm i.d.), filled (from bottom to top) with glass wool, deactivated silica gel (10% milli-Q water, 3 cm) and water-free sodium sulfate, that had been prewashed with 5 ml heptane. The extract was eluted with 10 ml of heptane. The concentration of the eluate to a volume of 0.5 ml was again performed with a rotational vacuum concentrator. PAH-Mix 9 deuterated standards (100 ng µl^−1^ of each component in cyclohexane) were obtained from Dr. Ehrenstorfer GmbH (Augsburg, Germany) and utilized to prepare an internal standard mix solution with a known concentration. The internal standard was added to each sample before extraction.

The final concentrated extracts were analyzed with gas chromatograph (Trace 1300) mass spectrometry (ISQ LT) (GC–MS, Thermo Scientific). A Rxi^®^-5 ms Crossbond^®^ 5% diphenyl and 95% dimethyl polysiloxane fused capillary column (30 m × 0.25 mm ID × 0.25 μm film thickness) from Restek (USA) was used with helium as the carrier gas at a constant flow rate of 1 ml min^−1^. The GC oven temperature was programmed to ramp from 75 °C (hold time 0.5 min) to 245 °C at 25 °C·min^−1^, then to 300 °C at 4 °C·min^−1^ (hold time 1 min). The injector and detector temperatures were 310 °C. The mass spectra were acquired in the electron ionization mode, while the selected ion monitoring (SIM) mode was carried out with the molecular ions selective for individual PAHs. The limits of quantification (LOQ) depending on individual PAHs ranged from 0.0002 to 0.3110 ng l^−1^ (C_free_) and from 0.1 to 0.7 µg kg^−1^ (C_tot_) for PAH concentrations and were obtained from three times the limit of detection (LOD).

### Ecotoxicological bioassays

Tests on the Collembola *Folsomia candida* were performed following the Organization for Economic Cooperation and Development (OECD) 232 method. The test endpoints were the mortality and reproduction of *F. candida*. The 10 randomly selected individuals collected from a synchronous culture (aged 10–12 days) were placed in the test vessel containing 30 g of soil sample. During the test, the organisms were fed dried yeast at an amount of 10 mg per vessel. The test vessels were incubated at a temperature of 20 ± 1 °C, with an illuminance of 400–800 lux and a 16/8 photoperiod (day/night). The soil moisture, set to 40–60% water holding capacity, was monitored once a week. The tests were performed in three replicates per test sample. After 28 days, the individuals were separated from the test vessel to determine the number of adult and juvenile individuals. The organisms were counted manually based on photos taken with a digital camera.

To evaluate the effect of the samples on *Lepidium sativum* plants, a Phytotoxkit test was performed. The evaluation parameter was root growth inhibition after three days of exposure to biochars in the solid and water phase. The liquid phase was prepared according to the EN 12457-2 protocol^29^. The soil samples were mixed with deionized water at a proportion of 1:10 (w:v, soil:water) and were subsequently shaken in a rotary shaker at 10 rpm. The obtained extract was filtered through filters with a porosity of 0.45 µm and the derived solution was used for further testing.

The evaluation of the extract’s toxicity to *Aliivibrio fischeri* was performed based on Microtox^®^ tests with a Microtox M500 analyzer. The luminescence inhibition was determined after 15 min of exposure of the extracts to *A. fischeri*. Microtox Omni software was employed to analyze the results. The bioassays were carried out in 6 replicates. The analysis and length measurements were carried out using Image Tool 3.0 software.

### Statistical analysis

All analyses were carried out with at least three replications. Each value represents the mean ± SD (n = 3). An analysis of variance (ANOVA) and the post-hoc Tukey test (Statistica 13.1) were conducted to assess the significance of the differences (*p* < 0.05) between the compared mean values.

## Results

### Physicochemical properties

The physicochemical properties are presented in Table 1. H500 and H700 were very similar in pH, while H600 had significantly lower values. The pH of the biochars was mostly basic. The differences in DW between the investigated biochars were not significant and amounted to approximately 95 ± 0.3%. The ash content increased with temperature. These changes were significant (*p* < 0.05; except for H600) and were 2.9–3.5 times higher than in the raw material.

**Table 1:** Physicochemical properties of obtained biochars from *Hermetia illucens* puparia.

	Puparia	H500	H600	H700
pH	–	10.310 ± 0.078^b^	9.280 ± 0.070^a^	10.577 ± 0.185^b^
Dry weight (%)	84.328 ± 3.588^a^	95.054 ± 0.241^b^	95.735 ± 0.626^b^	95.308 ± 0.389^b^
Ash (% DW)	12.528 ± 0.406^a^	37.178 ± 0.238^b^	41.739 ± 6.016^bc^	44.399 ± 0.696^c^
VS (% DW)	87.472 ± 0.287^c^	62.822 ± 0.168^b^	58.261 ± 4.254^ab^	55.601 ± 0.492^a^
H_2_O adsorption surface area (m^2^ g^−1^)	–	121.69 ± 0.68^b^	118.24 ± 0.68^b^	113.48 ± 2.61^a^
N_2_ BET surface area (m^2^ g^−1^)	–	1.972 ± 0.119^a^	12.103 ± 0.110^c^	4.666 ± 0.114^b^
N_2_ BET adsorption pore width (nm)	–	22.500	8.468	18.772
Pore volume less than 332.9 nm diameter (cm^3^ g^−1^)	–	0.011094	0.025623	0.021897
Total C (% DW)	39.430 ± 0.050^a^	40.635 ± 0.125^b^	40.635 ± 0.295^b^	45.505 ± 0.755^c^
Total H (% DW)	5.685 ± 0.065^d^	1.675 ± 0.025^c^	1.125 ± 0.075^b^	0.825 ± 0.005^a^
Total N (% DW)	8.215 ± 0.075^c^	7.235 ± 0.025^b^	5.600 ± 0.040^a^	5.750 ± 0.120^a^
Total S (% DW)	0.725 ± 0.001^b^	0.685 ± 0.000^a^	0.735 ± 0.001^c^	0.790 ± 0.002^d^
Total O* (% DW)	33.417 ± 0.091^d^	12.592 ± 0.125^c^	10.166 ± 0.411^b^	2.731 ± 0.880^a^
H:C	0.144 ± 0.002^d^	0.041 ± 0.000^c^	0.028 ± 0.002^b^	0.018 ± 0.000^a^
O:C	0.848 ± 0.001^d^	0.310 ± 0.004^c^	0.250 ± 0.012^b^	0.060 ± 0.020^a^

Different letters indicated statistically significant differences (Tukey’s test, *p* < 0.05). *DW* Dry weight, *BET* Brunauer–Emmett–Teller surface area.

*Calculated from the sum of ash, C, H, N, S assuming that the tested material consists only of these components.

The SSA measured by water vapor was the highest for H500 and the lowest for H700 (Table 1). In contrast, the BET SSA measured by N_2_ adsorption demonstrated low SSA values in the range of 2–12 m^2^ g^−1^ and no linear dependency with temperature (SSA was highest for H600). H600 had the smallest pore width and H500 the largest, with H700 in the middle (Table 1).

The content of C, H, N, O and S for H500, H600 and H700 were in the ranges 40.635–45.505%, 1.675–0.825%, 7.235–5.600%, 13.277–3.521% and 0.685–0.790%, respectively (Table 1). The ratio of H/C decreased significantly with a higher temperature. Furthermore, the ratio of O/C also decreased (*p* < 0.05) (Table 1).

**Figure 2:**
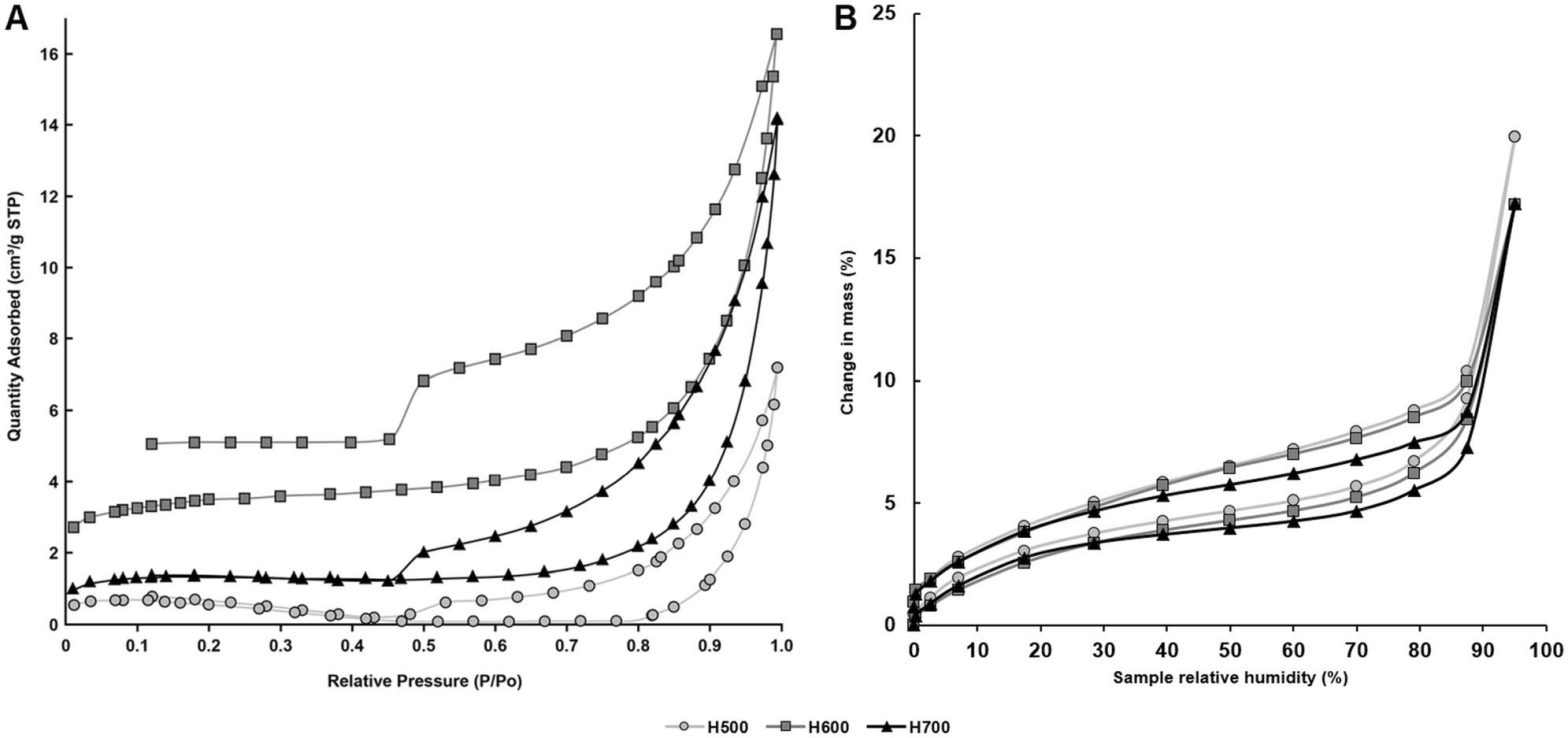
Adsorption–desorption isotherms for: (**A**) water vapor and (**B**) nitrogen gas for biochars obtained from *Hermetia illucens* puparia.

Figure 2A shows the adsorption–desorption isotherms of water vapor obtained for biochars. The isotherms were comparable to type IVa (according to the International Union of Pure and Applied Chemistry, IUPAC), although the plateau at a higher water vapor pressure was not observed^30^, which was corroborated with greater details from insights gained from N_2_ sorption. The shape of the hysteresis loop indicated the existence of small slits in the surface of the material^30^. Figure 2A also suggests the existence of quite uniform mesopores.

The course of the N_2_ adsorption–desorption curves resembled type IV adsorption (IUPAC classification) (Fig. 2B) and the hystereses in all cases were most similar to the H3 type. A sharp step-down threshold occurred between 0.4 and 0.5 P/Po.

### SEM microphotography

**Figure 3:**
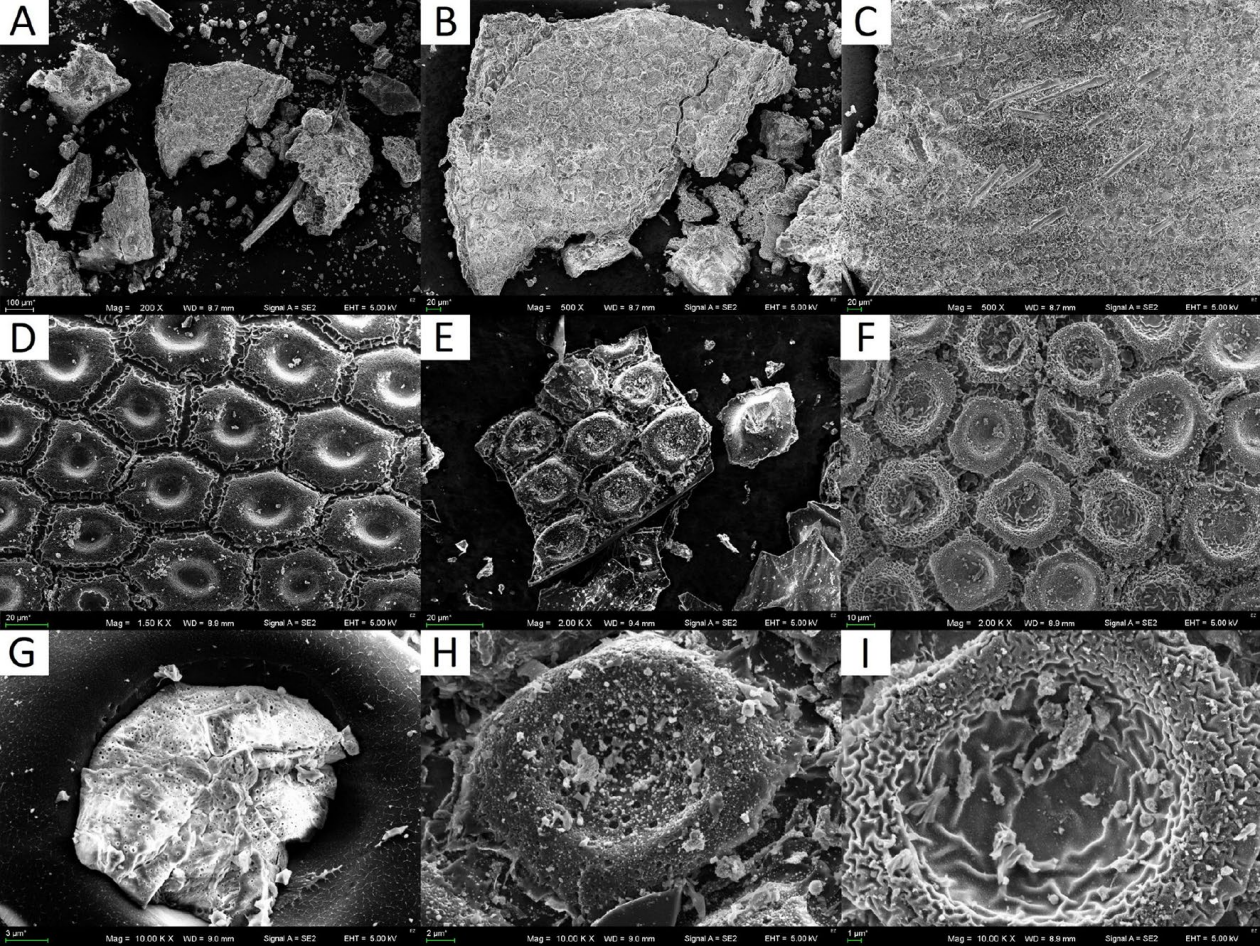
SEM photographs of *Hermetia illucens* puparia in different magnifications: (**A**) 200×, (**B**) 500×, and (**C**) 500×. SEM photographs of biochars obtained from *Hermetia illucens* puparia: (**D**) H500 at 1500× and (**G**) 10,000×; (**E**) H600 at 2000× and (**H**) 10,000×; (**F**) H700 at 2000× and (**I**) 10,000×.

**Figure 4:**
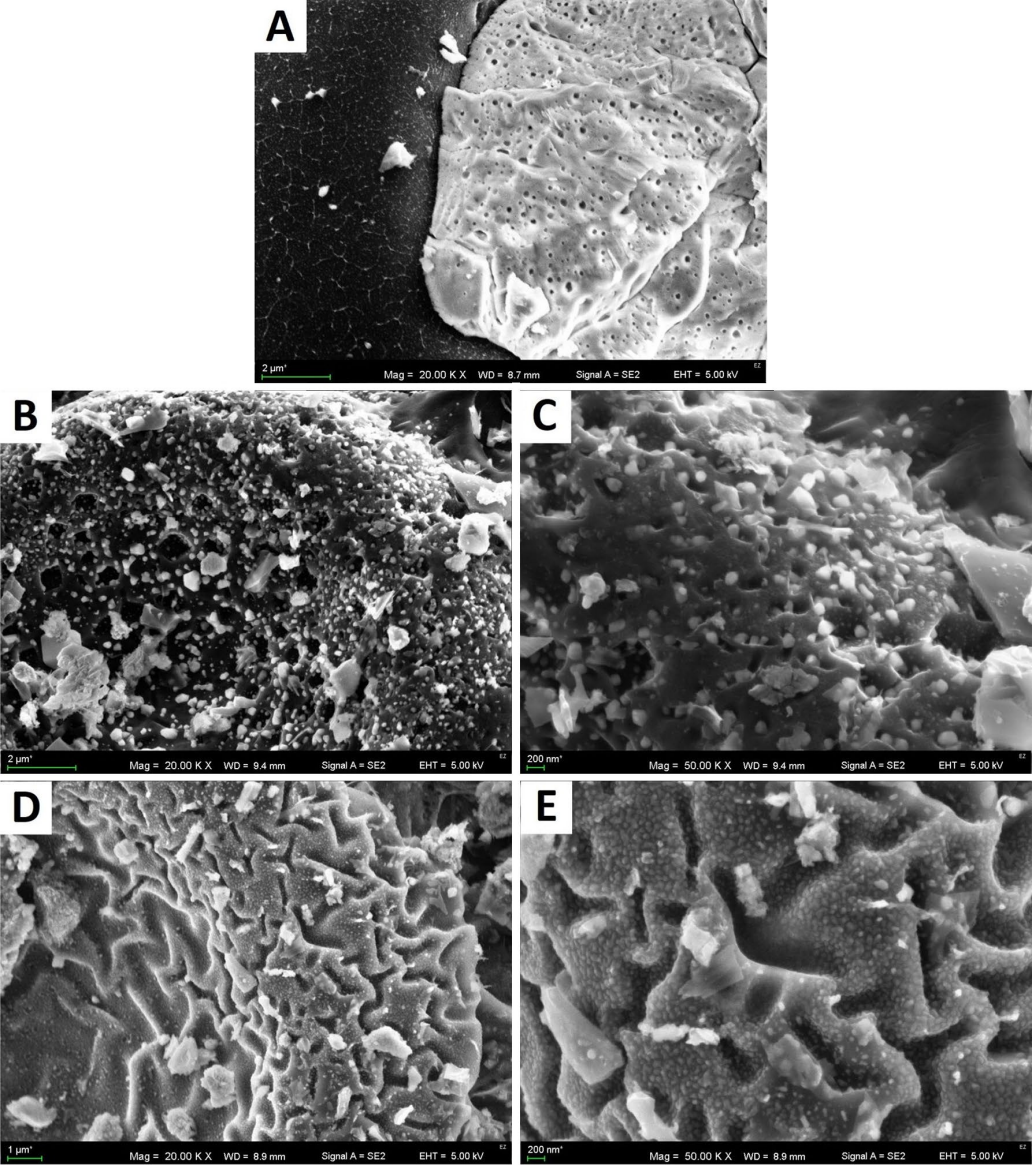
SEM photographs of biochars produced from *Hermetia illucens* puparia: (**A**) H500 (20,000×); (**B**) H600 (20,000×); (**C**) H600 (50,000×); (**D**) H700 (20,000×); (**E**) H700 (50,000×).

**Figure 5:**
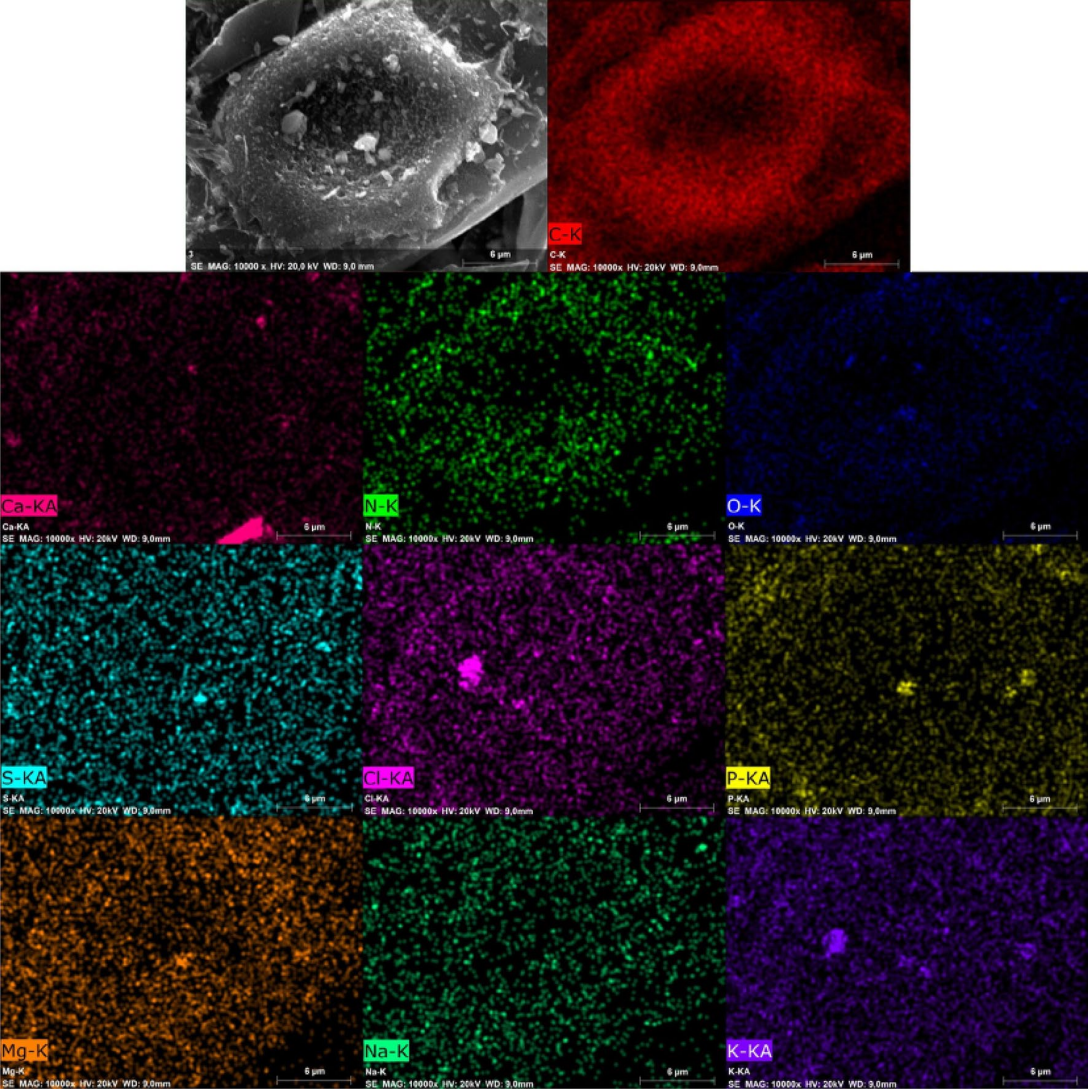
EDX surface maps of selected elements (magnification 10,000×) in H600 biochar. Top left image showed a SEM image of the sample.

Figure 3A–C shows SEM images of puparia. The internal sides of the puparia were characterized by irregular cells (Fig. 3B). From the outside, the presence of hairs can be observed (Fig. 3C). After pyrolysis, the hidden organization of the puparia-building cells becomes visible, which resembles a honeycomb structure (Fig. 3D–F). Under a magnification of 10,000× (Fig. 3G–I), details of the pores can be observed. The surface of H500 was smooth, and no pores were seen, while the central depression of the honeycomb structure was filled with ash (a bright material) (Fig. 3G). H600 had a well-developed porous structure with different pore sizes (Fig. 3H). H700 had a strongly wrinkled surface with no clearly visible pores. Under a higher magnification of 20,000× and 50,000× (Fig. 4), there were still no pores visible on the surface of the H500 biochar. In contrast, numerous pores partly filled with ash were seen in the H600 sample, but again, no pores were visible in the H700. Figure 5 shows the distribution of selected elements in the H600 material, chosen as the most interesting due to the visible pore structure. The elements Ca, Cl, K, Mg, O, P and S form distinct clusters on the surface, which correspond to the places where ash was present. In addition, the EDX map demonstrated that N was evenly distributed on the carbonized sample surface (Fig. 5).

### Elements content

In the biochar samples and puparia, 27 elements were determined (Table 2). Three patterns in the changes of element concentrations can be distinguished depending on the pyrolysis temperature. In the first group, the concentrations of Al, As, Ba, Co, Cr, Fe, K, Mg, Mo, and Na rose in the following order: H600 < H500 < H700 (Table 2). In the second group, those of Cd, Ni, and Se increased in the following order: H600 < H700 < H500. In general, in both groups of elements, the lowest concentration of a given element occurred at a temperature of 600 °C. The third group were elements whose concentrations increased with the increasing pyrolysis temperature: Ca, Cu, Mn, P, S, and Sr (i.e., H500 < H600 < H700). Pb was the only element whose concentration decreased with the rising pyrolysis temperature. For elements such as Be, Ga, Hg, Li, Sc, and V, changes in their content were not significant (*p* < 0.05) in any of the investigated materials.

**Table 2:** The concentration of elements in puparia and biochars produced form *Hermetia illucens* puparia from three different pyrolysis temperatures (500, 600, 700 °C) in mg kg^−1^; for elements: Ca, Fe, K, Mg, Mn, Na, P and S (in italics) concentrations was given in g kg^−1^.

	Al	As	Ba	Be	*Ca*	Cd	Co	Cr	Cu	*Fe*	Ga	Hg	*K*	Li	*Mg*	*Mn*	Mo
Puparia	197.63 ± 2.02a	61.82 ± 0.56d	34.45 ± 0.07c	0.01 ± 0.00a	*119.77* ± *0.15d*	1.88 ± 0.11a	0.55 ± 0.06a	6.92 ± 0.23b	51.53 ± 1.00b	*7.60* ± *0.02d*	23.69 ± 15.79a	0.30 ± 0.15a	*220.30* ± *0.30a*	23.70 ± 3.64a	*153.33* ± *0.11d*	*0.11* ± *0.00c*	54.21 ± 0.05d
H500	511 ± 2.26c	41.63 ± 0.40b	23.68 ± 0.09a	0.11 ± 0.08a	*76.45* ± *0.04a*	3.55 ± 0.18d	0.65 ± 0.16a	7.33 ± 0.29b	42.96 ± 0.64a	*6.02* ± *0.03b*	6.23 ± 3.33a	0.33 ± 0.21a	*26.91* ± *0.02c*	18.81 ± 3.47a	*11.10* ± *0.04b*	*0.87* ± *0.00a*	38.13 ± 0.14b
H600	352.70 ± 2.99b	38.07 ± 0.48a	23.43 ± 0.22a	0.04 ± 0.02a	*86.15* ± *0.10b*	2.24 ± 0.05b	0.47 ± 0.05a	5.26 ± 0.12a	43.91 ± 0.95a	*5.06* ± *0.04a*	14.77 ± 5.23a	0.23 ± 0.17a	*25.42* ± *0.34b*	18.49 ± 1.27a	*9.32* ± *0.10a*	*1.06* ± *0.00b*	34.66 ± 0.17a
H700	601.20 ± 2.20d	49.75 ± 0.75c	32.41 ± 0.18b	0.05 ± 0.00a	*99.76* ± *0.10c*	3.06 ± 0.06c	1.00 ± 0.02b	7.40 ± 0.58b	62.30 ± 0.30c	*7.46* ± *0.04c*	6.03 ± 1.12a	0.30 ± 0.21a	*31.76* ± *0.19d*	20.94 ± 4.22a	*11.33* ± *0.07c*	*1.14* ± *0.00d*	44.66 ± 0.01c

	*Na*	Ni	*P*	Pb	*S*	Sc	Se	Sr	V	Zn							
Puparia	*3.60* ± *0.04a*	6.82 ± 0.09b	*25.19* ± *0.02a*	11.98 ± 0.45c	*7.25* ± *0.01b*	1.04 ± 0.35a	1.93 ± 1.45a	164.47 ± 3.68c	6.17 ± 1.98a	333.23 ± 0.85a							
H500	*4.88* ± *0.02c*	9.59 ± 0.17c	*38.51* ± *0.07b*	28.60 ± 1.87d	*6.85* ± *0.00a*	0.74 ± 0.33a	8.08 ± 0.79b	49.11 ± 0.27a	3.11 ± 1.69a	352.67 ± 0.35b							
H600	*3.87* ± *0.06b*	4.58 ± 0.06a	*39.00* ± *0.06c*	3.73 ± 1.23b	*7.35* ± *0.01c*	0.62 ± 0.15a	1.75 ± 0.69a	58.71 ± 0.55b	2.97 ± 2.17a	354.70 ± 0.26c							
H700	*5.71* ± *0.04d*	6.67 ± 0.08b	*57.78* ± *0.13d*	0.58 ± 0.35a	*7.90* ± *0.02d*	0.73 ± 0.09a	1.94 ± 0.57a	62.85 ± 0.27b	5.55 ± 1.82a	563.87 ± 1.00d							

Means ± SD (n = 3). Different letters indicated statistically significant differences (Tukey’s test, *p* < 0.05). Statistic test was done for all materials for each element separately.

### FT-IR analysis

**Figure 6:**
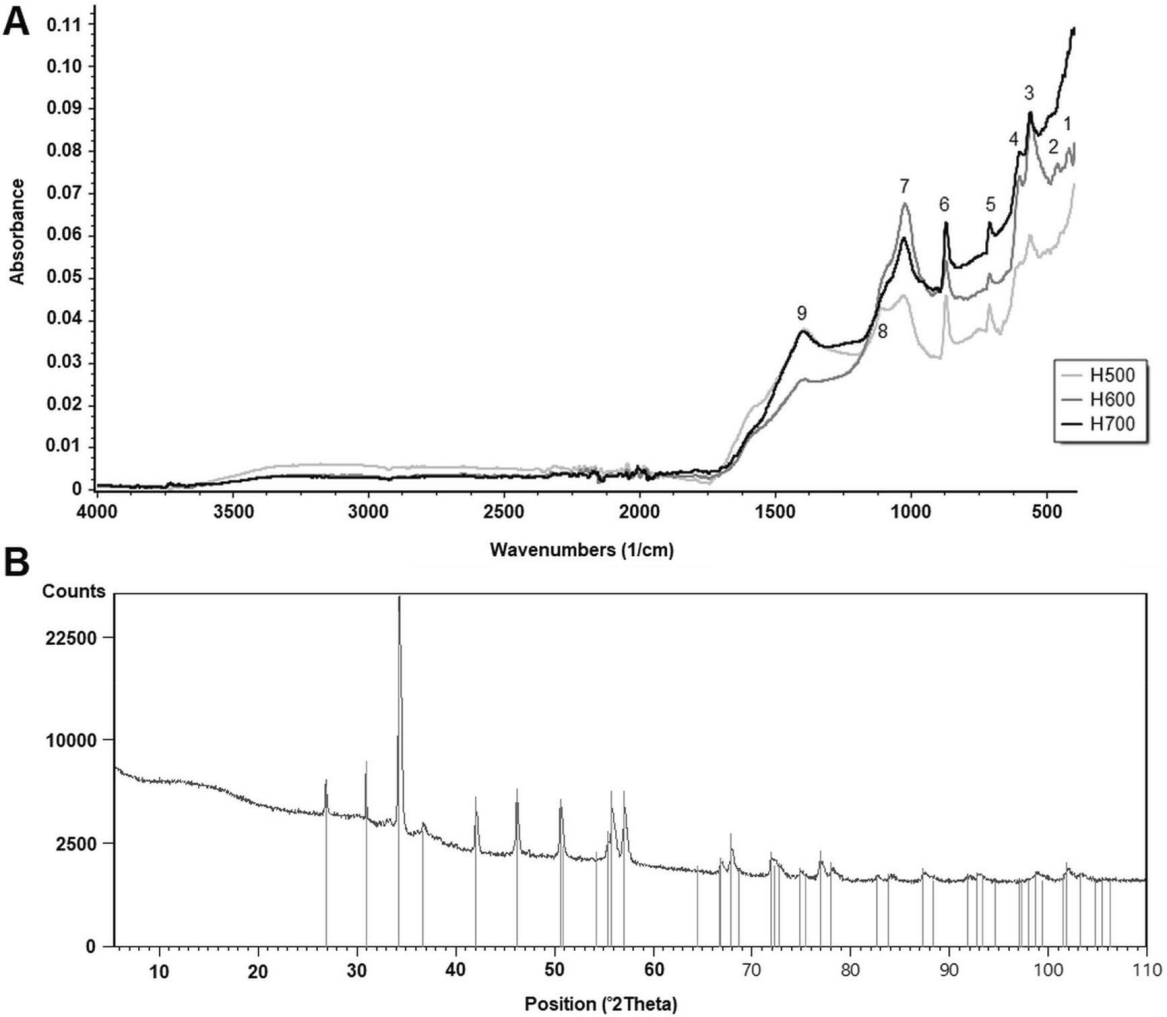
(**A**) FT-IR spectrum of biochars (H500–H700) obtained from *Hermetia illucens* puparia and (**B**) XRD pattern obtained for H600 biochar.

Figure 6A presents the FT-IR spectra for the studied biochars. The materials obtained in this study had a low number of characteristic bands placed mainly in the region of 800–1800 cm^−1^ (Table 3). Moreover, the biochars had only one common band with the raw material, which was placed around 870–890 cm^−1^ and corresponded with aromatic C–H ring stretching in a saccharide ring^21^ or C–H bending and C=C bending in alkene^8^. All biochars showed bands around 710 and 872 cm^−1^, but only H500 had a weak band at 1108 cm^−1^, which, according to Qambrani et al.^31^, corresponded to symmetric C–O stretching, e.g., in cellulose and hemicellulose. Interestingly, H600 had the highest intensity of a 1024 cm^−1^ band, which corresponded to C–O–C asymmetric stretching in a saccharide ring, but the lowest intensity of a band at 1390 cm^−1^ (aromatic C=C, aliphatic α-C–H_2_, phenolic O–H bending^31^, CH bending, and CH_3_ symmetric deformation^20^.

**Table 3:** Fourier-transform infrared spectroscopy (FT-IR) spectral characteristic of biochars obtained from *Hermetia illucens* puparia compared with literature data. Functional groups vibrations determined according to Waśko et al.^8^, Zazycki et al.^20^, Qambrani et al.^32^.

Functional group	Puparia (from^8^)	H500	H600	H700	Peak No
–	–	–	418	–	1
Si–O–Si	–	–	459	–	2
–	–	560	558	561	3
–	–	–	600	598	4
aromatic C–H out of plane deformation	–	711	710	710	5
aromatic C–H ring stretching (saccharide ring); C–H bending (β-glucosidic linkage), C=C bending	892	872^z^	872^z^	871^z^	6
C=C bending (alkene disubstituted (*trans*))	950	–	–	–	–
C–O stretching (primary alcohol)	1007	–	–	–	–
C–O–C asymmetric stretching in phase ring (saccharide ring)	–	1028^z^	1024^z^	1027^z^	7
C–O stretching (primary alcohol)	1069	–	–	–	–
symmetric C–O stretching	–	1108	–	–	8
C–O asymmetric stretching (aliphatic ether)	1114	–	–	–	–
C–O asymmetric stretching (aliphatic ether)	1152	–	–	–	–
C–N stretching (aromatic amine)	1309	–	–	–	–
C–H bend, CH_3_ symmetric deformation	1377	–	–	–	–
aromatic C=C, aliphatic α-C–H_2_, phenolic O–H bending or CH bending and CH_3_ symmetric deformation	–	1393^z^	1390^z^	1396^z^	9
N–H band and C–N stretch (Amide II)	1550	–	–	–	–
C=O sec amide stretch (Amide I)	1617	–	–	–	–
C=O sec amide stretch (Amide I)	1654	–	–	–	–

^z^Bands common with biochar made from shrimp (*Penaeus brasiliensis*) chitin (Zazycki et al.^20^).

### X-rays diffraction

Figure 6B presents an example of the X-ray diffraction patterns of the H600 biochar, which were common to all the obtained biochars. A sharp peak around 26.6 2θ° was distinctive. Besides, for *H. illucens* biochars, the major peaks were found at 34.2°, 42.2°, 46.1°, 50.8°, 55.0° and 56.0° respectively. The diffraction profile showed the presence of calcite (CaCO_3_) (based on the International Centre for Diffraction Data (ICDD) database PDF4 + 2018).

### Adsorption of Ni, Cd, and Pb

**Figure 7:**
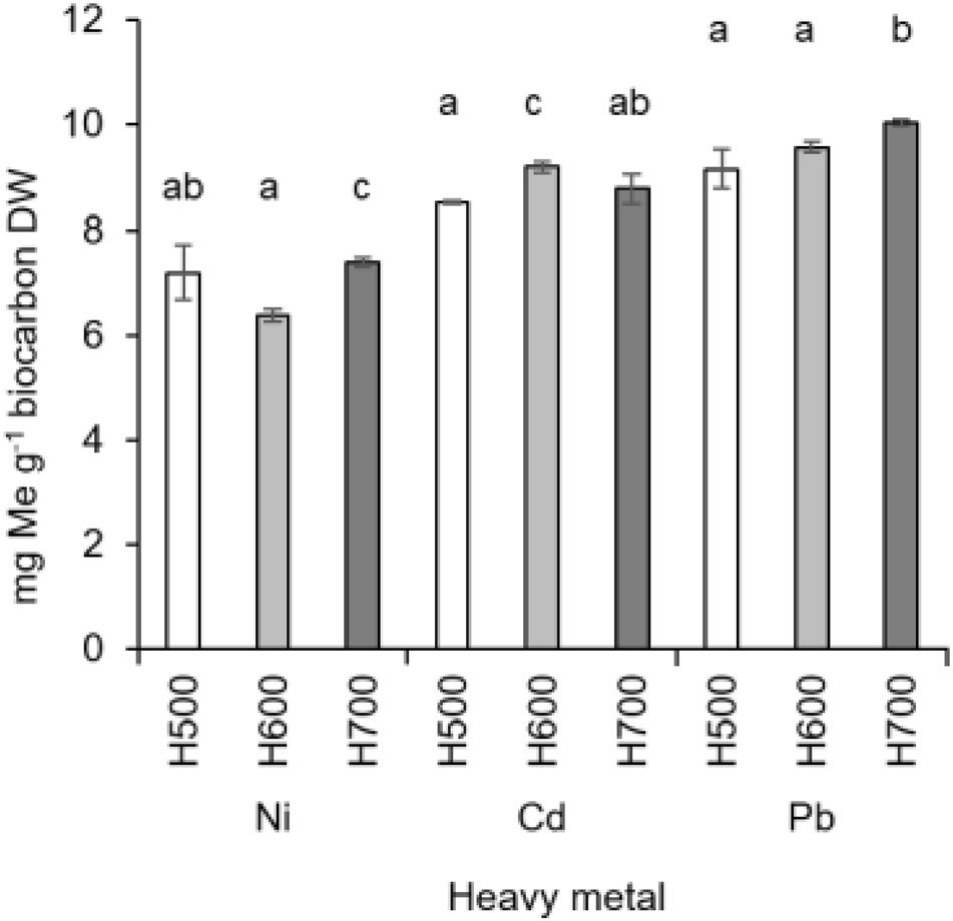
Adsorption of selected heavy metals on biochars (H500, H600, H700) obtained from *Hermetia illucens* puparia. Means ± SD (n = 3). Different letters indicated statistically significant differences (Tukey’s test, *p* < 0.05).

The investigated biochars were characterized by the highest adsorption ability for Pb (the average for all the materials together was 9.6 ± 0.4 mg Pb g^−1^) (Fig. 7). The next highest was Cd (8.8 ± 0.3 mg Cd g^−1^) and the lowest adsorption was identified for Ni (6.9 ± 0.5 mg Ni g^−1^) (Fig. 7). The pyrolysis temperature affected the adsorption abilities of different metals. The H700 biochar had a significantly higher sorption capacity for Ni (7.38 ± 0.09 mg Ni g^−1^) than the other biochars pyrolyzed at lower temperatures. In turn, the highest amount of Cd was absorbed by the H600 biochar (*p* < 0.05) and it was 9.19 ± 0.10 mg Cd g^−1^. The adsorption capacity of Pb increased with the pyrolysis temperature, so H700 was characterized by the highest amount of adsorbed Pb ions (10.05 ± 0.06 mg Pb g^−1^).

### PAHs content

For all the biochars, naphthalene (NAP) had the highest contribution to the total sum of PAHs and ranged from 49.5 to 59.6% (Table 4). In H500 and H700, acenaphthylene (ACY) was also widespread (19.2–25.0%), as was phenanthrene (PHE) in H600 (17.3%). The content of benzo[a]pyrene (BaP), which is known for its carcinogenic properties, decreased with the pyrolysis temperature. The C_free_ PAH with the highest contribution was also NAP (90.0–92.4% of total sum). In the second place in each material was acenaphthene (ACE), with a 2.8–3.6% share in the total sum, and in the third place were fluorene (FLO) in both H500 and H700 (2.6–2.7%) and anthracene (ANT) in H600 (1.7%) (Table 4).

**Table 4:** Polycyclic aromatic hydrocarbons (PAHs) content in biochars produced from *Hermetia illucens* puparia.

PAH	Total content C_tot_ (µg kg^−1^)			Freely dissolved in water extract C_free_ (ng l^−1^)		
Material	H500	H600	H700	H500	H600	H700
Naphthalene (NAP)	119.0 ± 10.2^ab^	100.1 ± 11.7^a^	125.0 ± 10.0^b^	65.0 ± 4.4^c^	43.1 ± 2.0^b^	54.6 ± 3.6^a^
Acenaphthylene (ACY)	60.0 ± 4.2^c^	14.9 ± 4.2^a^	47.7 ± 3.5^b^	0.26 ± 0.01^c^	0.33 ± 0.02^a^	0.43 ± 0.03^b^
Acenaphthene (ACE)	21.1 ± 1.7^b^	4.6 ± 0.3^a^	24.0 ± 1.3^c^	2.58 ± 0.22^c^	1.32 ± 0.10^b^	2.04 ± 0.11^a^
Fluorene (FLO)	6.1 ± 0.5^b^	4.7 ± 0.3^a^	6.8 ± 0.5^b^	1.87 ± 0.16^b^	0.59 ± 0.04^b^	1.64 ± 0.10^a^
Phenanthrene (PHE)	22.5 ± 2.1^a^	29.1 ± 2.4^b^	28.6 ± 1.8^b^	1.24 ± 0.14^c^	0.39 ± 0.03^b^	0.94 ± 0.07^a^
Anthracene (ANT)	2.9 ± 0.3^b^	4.9 ± 0.5^c^	1.8 ± 0.1^a^	0.61 ± 0.05^a^	0.78 ± 0.05^a^	0.54 ± 0.04^b^
Fluoranthene (FLA)	0.2 ± 0.0^a^	2.4 ± 0.2^b^	3.9 ± 0.3^c^	0.10 ± 0.01^b^	0.03 ± 0.00^c^	0.13 ± 0.01^a^
Pyrene (PYR)	0.4 ± 0.0^a^	2.9 ± 0.2^b^	4.4 ± 0.4^c^	0.17 ± 0.02^b^	0.07 ± 0.00^c^	0.25 ± 0.02^a^
Bezno(a)anthracene (BaA)	1.0 ± 0.1^b^	0.1 ± 0.0^a^	0.2 ± 0.0^a^	0.01 ± 0.00^a^	0.00 ± 0.00^b^	0.01 ± 0.00^a^
Chrysene (CHR)	1.0 ± 0.1^c^	0.8 ± 0.1^b^	0.2 ± 0.0^a^	0.010 ± 0.001^a^	0.010 ± 0.001^b^	0.021 ± 0.002^a^
Bezno(b)fluoranthene (BbF)	1.0 ± 0.0^a^	0.6 ± 0.1^a^	4.5 ± 0.4^b^	0.0058 ± 0.0000^a^	0.020 ± 0.002^b^	0.017 ± 0.001^c^
Benzo(k)fluoranthene (BkF)	0.5 ± 0.1^b^	0.3 ± 0.0^a^	0.6 ± 0.1^c^	0.0029 ± 0.0000^a^	0.003 ± 0.000^b^	0.004 ± 0.000^ab^
Benzo(a)pyrene (BaP)	1.6 ± 0.0^c^	1.4 ± 0.1^b^	0.4 ± 0.0^a^	0.0019 ± 0.0000^a^	0.013 ± 0.001^b^	0.011 ± 0.001^c^
Indeno(1,2,3-cd)pyrene (IcdP)	0.4 ± 0.0^b^	0.8 ± 0.1^c^	0.2 ± 0.0^a^	0.0010 ± 0.0000^a^	0.001 ± 0.000^b^	0.001 ± 0.000^a^
Dibenzo(a,h)anthracene (DahA)	2.1 ± 0.2^b^	0.2 ± 0.0^a^	0.1 ± 0.0^a^	0.0004 ± 0.0000^a^	0.001 ± 0.000^b^	0.001 ± 0.000^b^
Benzo(g,h,i)perylene (BghiP)	0.4 ± 0.0^c^	0.2 ± 0.0^b^	0.1 ± 0.0^a^	0.0028 ± 0.0000^a^	0.0030 ± 0.0000^a^	0.0030 ± 0.0000^a^
Sum	240.2 ± 31.8^b^	168.0 ± 25.0^a^	248.6 ± 32.2^b^	71.9 ± 16.2^c^	46.7 ± 10.7^b^	60.7 ± 13.6^a^

Means ± SD. Different letters indicated statistically significant differences (Tukey’s test,* p* < 0.05). PAHs concentrations were statistically tested between biochars for each PAH in each group (total or water extract) separately.

In all the biochars, NAP also had the highest contribution to the total sum of PAHs in water extract (C_free_ PAH), which was 90.0–92.4% (Table 4). The next highest contributor in each material was acenaphthene (ACE), with a 2.8–3.6% share in the total sum. Fluorene (FLO) was also detected in all the biochars, in the concentration range 1.7–2.7%. Phenanthrene (PHE) was identified only in H500 and H700 in the concentration range 1.5–1.7%. Anthracene (ANT) was present only in H600 (1.7% of the total sum). The share of other PHAs in the total sum was below 1% (Table 4).

### Ecotoxicology tests

**Figure 8:**
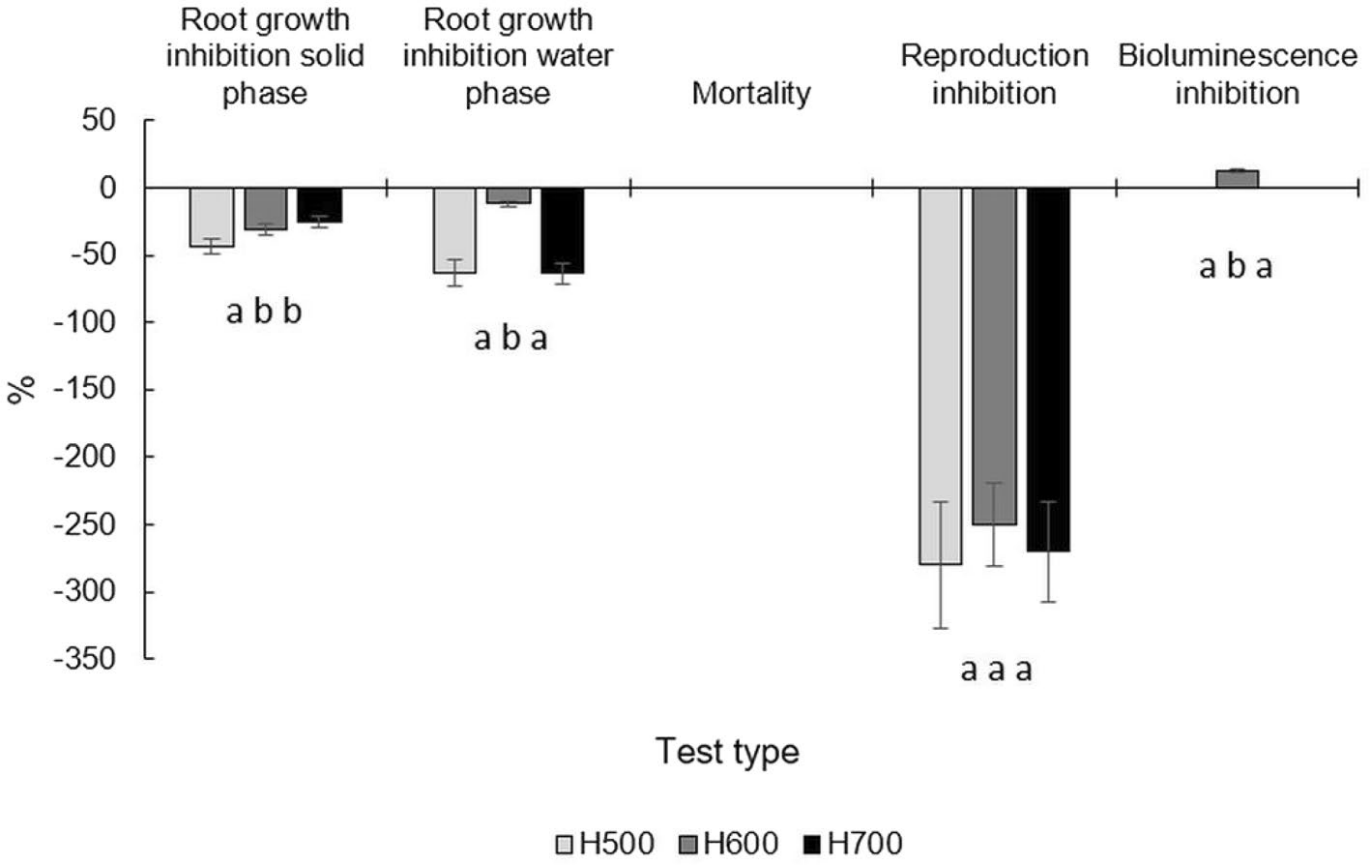
Ecotoxicology tests results for biochars produced from *Hermetia illucens* puparia. The effects of investigated biochars on: *Lepidium sativum* root growth inhibition in solid phase and in water extracts, mortality and reproduction inhibition of *Folsomia candida* and bioluminescence inhibition of *Aliivibrio fischeri*. Means ± SD (n = 3). Different letters indicated statistically significant values (Tukey’s test, *p* < 0.05).

All the tested materials showed a stimulation effect on the growth of *L. sativum* roots, which was significantly higher for H500 (Fig. 8). This trend decreased with the pyrolysis temperature (Fig. 8). Root growth stimulation also occurred for water extracts regardless of the pyrolytic temperature—this was the highest and similar for both H500 and H700 (63.5% on average), while for H600, it reached only 12.0 ± 1.9% (Fig. 8). Biochars had no effect on *F. candida* mortality but showed a high influence on the stimulation of their reproduction (Fig. 8). Only H600 displayed an inhibitory effect on *A. fischeri* bioluminescence (Fig. 8).

## Discussion

The pH of the biochars was mostly basic due to thermal dehydration reactions and the formation of ash^32,33^. However, Hossain et al.^34^ proved that it strongly depends on the initial pH of the raw material. An inversely proportional tendency to the temperature was observed for the volatile solid (VS) content, and this phenomenon is well known^33^.

Investigated biochars had low SSA for this type of material. In general, the average SSA has the tendency to increase with the pyrolysis temperature^31^. However, a decrease in SSA can be connected to increasing ash content due to the temperature of the pyrolysis, which by clogging the pores reduces the SSA of the materials^33^, or by the sintering processes, which occurred more significantly when the temperature increased^35^. Sintering could be proven by SEM images at ×50,000 magnification (Fig. 4), which showed only wide slit-like structures on the surface of the H700 biochar in comparison to the H600 material, in which a porous structure could be seen.

Biochars produced from a single raw material may have SSAs in a wide range depending on the pyrolysis condition. Biochars obtained from *H. illucens* puparia were similar in this parameter to biochars from: orange peel (7.8–51.0 m^2^ g^−1^), palm bark (2.5 m^2^ g^−1^), peanut shells (3.1 m^2^ g^−1^), pine needle (0.7–19.9 m^2^ g^−1^), pine pitch (2.9–4.8 m^2^ g^−1^), pine wood shavings (1.8–4.8 m^2^ g^−1^), poultry litter (3.9 and 9.0 m^2^ g^−1^), soybeans stover (5.6 m^2^ g^−1^), swine solids and manure (4.1 and 5.7 m^2^ g^−1^), turkey litter (2.6 m^2^ g^−1^) and weaner manure (3.8 m^2^ g^−1^)^33^. Biochars produced from sewage sludges and biogas residues may also have an SSA in a similar range, as revealed by Stefaniuk and Oleszczuk^32^ and Zielińska et al.^33^.

The average pore width responsible for N_2_ adsorption in investigated materials was in the mesopore range (2–50 nm)^30^. Zielińska et al.^33^ showed an average pore size in the range of 5.7–14.0 nm in biochars from different sewage sludges. Stefaniuk and Oleszczuk^32^ proved that the pore size of some biochars from biogas residues may be in a similar range to that observed in this study.

The adsorption–desorption isotherms for N_2_ in the type IV materials occur in mesoporous materials and are characterized by condensation in pores but without achieving the saturation plateau^35^ (Fig. 2B). The H3 hysteresis occurs in non-rigid aggregates of plate-like particles or when the macropores are not completely filled with pore condensate^30^. This evidenced the slit-like pore shape characteristic of a carbon absorbent^33^. Moreover, the sharp step-down threshold between 0.4 and 0.5 P/Po pointed once again to partially blocked mesopores^30^.

The raw material (puparia) showed a similar content of C and H to that of the study by Waśko et al.^8^, with the one exception of N content, which may be connected to the differing diets of insects and with the fact, that in this research the puparia did not undergo any cleaning or pre-treatment. In general, the composition of *H. illucens* puparia was very similar to that of pure chitin of marine origin^21^. In the work of Zazycki et al.^21^, biochars from pure chitin had been produced by pyrolysis at 800 °C/60 min. They determined the C content to be as high as 81.30%, while the contents of H, N and O were 2.15%, 3.45%, and 13.10%, respectively. Similarly, Magnacca et al.^36^ ascertained the C content in biochars produced from marine chitin to be in the range 72.7–75.5% for materials pyrolyzed at 440 and 540 °C, but for material obtained at 294 °C the carbon content was very similar to that of the raw material (47.7%). In the cited research, the content of H and N for materials obtained at 440 and 540 °C was 3.5–2.8% and 8.3–8.0%, respectively. Biochars produced from *H. illucens* puparia had much lower contents of C and H but similar levels of N and O in comparison to the above mentioned research.

The biochars investigated in this study were similar in C and H content to: broiler litter (700 °C/60 min, 46% C, 1.4% H), chicken litter (620 °C/2 h, 41.5% C, 1.2% H), goat manure (400–800 °C/30 min, 42.7–43.6% C, 1.7–0.8% H), swine manure (400 °C/60 min, 41.8% C, 1.0% H), swine solid (700 °C/2 h, 44.1% C, 0.7% H) and turkey litter (700 °C/2 h, 44.8% C, 0.95% H)^31^. Elemental composition showed that the materials examined in this research can be located between biochars obtained from animal manures and pure marine chitin.

The sulfur content in biochars is of much less interest, as can be seen from the fact that the review paper by Qambrani et al.^31^ did not summarize its content. The works of Zazycki et al.^21^ and Magnacca et al.^36^ also did not investigate it. The S content in biochar produced from corn straw pyrolyzed at temperatures of 300–700 °C/6 h was in the range of 0.32–0.58%^37^. Biochars made from oak and corn stover (500–850 °C) had contents of S in the ranges of 0.15–0.17% and 0.61–0.80%^38^. Recently, Leng et al.^39^ reviewed the S content of biochars from different feedstock materials. Generally, in biochars made from plant materials, the S content was in the range of 0.015–0.550% but was the highest in biochars produced from sewage sludge (0.747–0.996%) and those from chicken manure were between. Thus, in terms of S content, biochars made from *H. illucens* puparia were the most similar to chicken manure biochars. In general, biochars derived from plant biomass and animal manures had a lower N content than the materials obtained in this study (up to 4.9% for pelletized poultry litter and 4.1% for pine needles), while O content differed in a wide range of values^31^. In this context, biochars derived from *H. illucens* puparia constitute a new and interesting material with naturally high N concentration. Biochar doped with heteroatoms, especially with N, deserves special attention. For example, it can be used as electrocatalytic materials in microbial fuel cells, lithium or zinc-air batteries, and supercapacitors^40^.

The decrease of H/C and O/C ratios with a higher temperature indicated an increase in the carbonization and aromatization of C–C bonds and decrease in the presence of oxygen functional groups due to dehydrogenation reactions in the biochars. The lowering of both parameters indicated the increased hydrophobicity of the obtained materials^32,33^. Biochars with low H/C values could be useful for long-term C sequestration in the soil due to their resistance to microbial degradation^33^.

Microphotography showed the well-preserved honeycomb structure of the biochars, which survived the pyrolysis conditions. This internal structure of the puparia was also visible under SEM magnification in the pure chitin extracted from it^8^. The smoothness of the biochar surface at lower temperatures, like in H500, has been previously observed by Pituello et al.^41^. Figure 3H showed that pore structure had developed for H600 but not for H700. This was why H600 had the highest N_2_ BET surface area (Table 1). A higher temperature caused the closing of previously present pores due to wrinkling and sintering. It is therefore probable that even better pore structure and a larger SSA could be obtained by adjusting the pyrolysis temperature more precisely.

Zhao et al.^42^ showed that the content of elements in biochars increased with the temperature of the production process. However, this was not always the case. Exceptions have been demonstrated for the contents of Cu and Mn, which were the highest at 500 °C, while the highest temperature used in this study was 600 °C^42^. These non-linear tendencies are often seen in the literature, and they result from various processes that have not yet been accurately characterized. Some authors supposed that different metals might be volatilized at higher temperatures in the process^41,42^. Biochars produced from *H. illucens* puparia were in general more abundant in micro- and macroelements, e.g., they had a higher content of Ca, Mg, Fe, Mn, Cu, and Zn than biochar from apple tree branches^42^. Similarly, the content of Zn in biochars from *H. illucens* puparia was higher than in materials produced from vineyard pruning residues^41^, as well as wood pellets and brushwood^43^. This indicated that there would be a high microelement supplementation value for plants if these biochars were employed as biofertilizer.

Biochars produced from *H. illucens* had higher concentrations of As and Cd than biochars from all plant and non-plant materials studied by Pituello et al.^41^ and Marmiroli et al.^43^. However, Pb and Ni were lower in comparison to biochars obtained from sewage sludge and municipal organic waste digestate, respectively^41^. The Cr content was also lowest in puparia-derived biochars in contrast to the above-mentioned materials.

The higher content of elements in comparison to plant biochars can be explained by the phenomenon of bioaccumulation that occurs in *H. illucens* during feeding*.* Some heavy metals, such as Cd and Zn in particular, are known to undergo bioaccumulation in this insect^13,44^. Proc et al.^45^ recently demonstrated the ability of *H. illucens* to bioaccumulate many more elements. In the puparia of *H. illucens* fed with non-spiked feed, the bioaccumulation of Ba, Ca, Cu, Fe, Ga, Hg, Mg, Mo, Mn, P, S, and Se were shown, with an especially high bioaccumulation factor obtained for Ca (12.02) and Mn (5.95)^45^.

FT-IR analysis of H500 indicated that the puparia used for pyrolysis may have been surface-contaminated with the substrate residues in which the larvae lived (the substrate was based on coconut fiber). At higher temperatures, this residue disintegrated, and the band disappeared. Puparia taken for the experiment were not cleaned purposefully. Washing them before pyrolysis would be unjustified for economic reasons in the conditions of a real company wanting to produce biochar from this type of waste product. A couple of bands presented in Table 3 (peaks no. 6, 7, and 9) were similar to the biochar produced from purified shrimp chitin^21^.

In the XRD spectrum, the peak at 26.6 2θ° represented a stacked graphitic basal phase at the plane and signified the crystallization of the carbon^46^. A high, thin peak indicates more crystallization than a broader, hill-like shape. The presence of calcite was also detected in biochars made from biogas residues^32^ and sewage sludges^33^. As compared to the diffraction patterns obtained for chitin extracted from *H. illucens* puparia^8^, no peaks ranging from 9 to 25 2θ° were observed in H600. In raw puparia, these peaks indicate an α-chitin crystal structure^8,21^. The disappearance of the crystalline structure already occurred at 600 °C in processed puparia and was further confirmed by the lack of amide I and amide II bands on the FT-IR spectrum (Fig. 6A). This is consistent with the observation of Zazycki et al.^21^.

Kılıç et al.^47^ reported that the adsorption capacity for biochar obtained from almond shells at 650 °C was 20 mg Ni g^−1^ (pH 6.0). Bogusz et al.^48^ showed Ni adsorption in the range of 16.6–34.2 mg Ni g^−1^ (pH 5.5) of biochars obtained from residues after biogas production. The range of Ni sorption on different biochars from plant origin and broiler litter was 1.17–19.80 mg Ni g^−1^^48^.

Biochar produced from *Miscanthus sacchariflorus* at 300–600 °C had an adsorption capacity for Cd at pH 7 in the range of 11.40–13.24 mg Cd g^−1^^49^. Biochar generated from manure at 200 and 350 °C had an even higher sorption: 31.9 and 51.4 mg Cd g^−1^ as reported by Xu et al.^50^. On the other hand, biochar from oak bark obtained at 400–450 °C and tested at pH 5 had a much lower adsorption capacity for this metal (5.4 mg Cd g^−1^)^51^ than biochars obtained from puparia.

The materials investigated in this study had a much higher adsorption capacity for Pb than biochars produced from pinewood and rice straw during hydrothermal liquefaction at 300 °C (which adsorbed 3.89 mg Pb g^−1^ and 1.84 mg Pb g^−1^ respectively)^52^. Other biochars from plant origin, like pine wood, pine bark, oak wood, and oak bark, pyrolyzed at 400–450 °C had an adsorption capacity for Pb in the range of 2.62 (oak wood) to 13.1 (oak bark) mg Pb g^−1^ (pH 5.0) as revealed by Mohan et al.^51^. A much higher adsorption was described by Lu et al.^53^ on biochar produced from sludge at 550 °C (30.9 mg Pb g^−1^; pH 5.0).

Cd and Pb adsorption on biochar surfaces mainly depends on cation exchange, surface complexation, precipitation, and, especially for Cd, on electrostatic interactions as well^54^. Sorption characteristics and responsible mechanisms strictly depend on the type of biochar and the raw material, as well as the pyrolysis conditions, in addition to the solution pH, and can vary widely^54^. The cited literature allows for describing biochar from *H. illucens* puparia as moderately adsorbent for the discussed heavy metals.

The total content of 16 US Environmental Protection Agency (EPA) PAHs did not exceed the recommended maximum concentration of 300 mg kg^−1^ DW^55^ and was considerably below this limit (168–249 µg kg^−1^ DW) (Table 4). NAP was the most abundant PAH within the obtained biochars. This results from a pyrolysis temperature greater than 500 °C, from which the free radical mechanism of PAH formation begins to predominate, resulting in the production of NAP as the most thermodynamically stable compound^56^. The content of Σ16 PAHs in water extract, which are bioavailable and mainly responsible for negative environmental effects^56^, was in an even lower range of 48–72 ng dm^−3^ (Table 4). Due to its low concentration and its ability to form strong bonds with the surface of biochar, this material acts as a sorption sink rather than a source of PAHs^56^.

Biochars from *H. illucens* puparia express no toxicity for *L. sativum* and model soil invertebrates, even despite the fact, that the tested materials contained a higher total PAH content than reported in Kołtowski and Oleszczuk^57^. *F. candida* is found in soils around the world and belongs to the Isotomidae family of the Collembolans. Egamberdieva et al.^58^ demonstrated the addition of biochar to soil-stimulated plant growth through the stimulation of rhizobacteria growth. Additionally, the increase in the reproduction rate of *F. candida* could also be connected with the stimulation of bacterial growth, which in turn could intensify the decomposition of organic matter on which *F. candida* feeds. Only one negative effect has been seen, and that was the inhibition of *A. fisheri* bioluminescence caused by H600 (Fig. 8). Due to the fact that in H600 the content of heavy metals and other elements was either lower or statistically insignificant than in the other tested biochars (Table 2), organic compounds were suspected of having an inhibition effect. Generally, the toxicity of extractable substances from biochar has not been a frequently discussed or studied topic, but certainly requires exploration due to the content of substances such as PAHs, dioxins, tars, furans, volatile organic compounds, and toxic heavy metals^57,59^. Intani et al.^59^ reported the toxicity of corn cob biochars on *L. sativum*, which affected its germination rate, shoot length, and fresh shoot weight. Kołtowski and Oleszczuk^57^ confirmed the high, medium, and very low toxicity of biochars produced from miscanthus, willow, and wheat straw, respectively on *A. fischeri* bioluminescence and *Daphnia magna* survival.

## Conclusions

Our results demonstrate how the non-standard management of waste from the dynamically growing insect breeding industry may be used for the production of biochar with new properties. This new biochar showed, in terms of most parameters, intermediate properties between biochars of plant origin and those produced from manure, sewage sludge, and biogas sludge. Despite the fact that its sorption properties were low, the higher content of nitrogen and numerous micro- and macroelements in comparison to biochars from plant biomass^31^ make it suitable for agricultural usage. The content of heavy metals was within (As, Cd, Mo, Zn) or lower (Cr, Hg, Ni) than the standard for biochars developed by the International Biochar Initiative^55^ and guarantees its safe use. Naphthalene was the most abundant PAH within the obtained biochars. The total content of 16 US EPA PAHs did not exceed the recommended maximum concentration of 300 mg kg^−1^ DW^55^ and was below this limit (168–249 µg kg^−1^ DW for total content and 48–72 ng l^−1^ for water extracts). Finally, the lack of toxicity, and moreover, the growth stimulation effect on *L. sativum* root and increased reproduction of *F. candida* indicate the remarkable properties of the produced biochars, which may find practical application in agriculture. For instance, these positive features, together with the extended surface area of obtained biochars, allow to indicate its potential use, especially as a carrier of fertilizers or beneficial bacteria. Very interesting would be the activation of this type of biochar, which should result in a much higher surface area and improve sorption properties. Due to their naturally occurring high N content, the investigation of their electronic properties, e.g., in the formulation of supercapacitor electrodes, also sets a potential direction for future research. However, the most important direction in the near future will probably be to investigate the properties of biochar obtained from the exoskeletons of other insect species as well as from their frass.

## Data Availability

Data will be made available on request from the corresponding author—Piotr Bulak.
